# Pyridinecarboxylic Acid Derivative Stimulates Pro-Angiogenic Mediators by PI3K/AKT/mTOR and Inhibits Reactive Nitrogen and Oxygen Species and NF-κB Activation Through a PPARγ-Dependent Pathway in *T. cruzi*-Infected Macrophages

**DOI:** 10.3389/fimmu.2019.02955

**Published:** 2020-01-09

**Authors:** Federico Nicolás Penas, Davide Carta, Ágata Carolina Cevey, María Jimena Rada, Azul Victoria Pieralisi, María Grazia Ferlin, María Elena Sales, Gerardo A. Mirkin, Nora Beatriz Goren

**Affiliations:** ^1^Departamento de Microbiología, Parasitología e Inmunología, Facultad de Medicina, Universidad de Buenos Aires, Buenos Aires, Argentina; ^2^Instituto de Investigaciones Biomédicas en Retrovirus y Sida (INBIRS), CONICET-Universidad de Buenos Aires, Buenos Aires, Argentina; ^3^Department of Pharmaceutical and Pharmacological Sciences, University of Padova, Padova, Italy; ^4^Centro de Estudios Farmacológicos y Botánicos (CEFyBO), CONICET-Universidad de Buenos Aires, Buenos Aires, Argentina; ^5^Instituto de Investigaciones en Microbiología y Parasitología Médica (IMPaM), CONICET-Universidad de Buenos Aires, Buenos Aires, Argentina

**Keywords:** new PPARγ ligand, PI3K/AKT/mTOR, NF-κB pathway, *Trypanosoma cruzi*, macrophages

## Abstract

Chagas disease is caused by *Trypanosoma cruzi* infection and represents an important public health concern in Latin America. Macrophages are one of the main infiltrating leukocytes in response to infection. Parasite persistence could trigger a sustained activation of these cells, contributing to the damage observed in this pathology, particularly in the heart. HP_24_, a pyridinecarboxylic acid derivative, is a new PPARγ ligand that exerts anti-inflammatory and pro-angiogenic effects. The aim of this work was to deepen the study of the mechanisms involved in the pro-angiogenic and anti-inflammatory effects of HP_24_ in *T. cruzi*-infected macrophages, which have not yet been elucidated. We show for the first time that HP_24_ increases expression of VEGF-A and eNOS through PI3K/AKT/mTOR and PPARγ pathways and that HP_24_ inhibits iNOS expression and NO release, a pro-inflammatory mediator, through PPARγ-dependent mechanisms. Furthermore, this study shows that HP_24_ modulates H_2_O_2_ production in a PPARγ-dependent manner. It is also demonstrated that this new PPARγ ligand inhibits the NF-κB pathway. HP_24_ inhibits IKK phosphorylation and IκB-α degradation, as well as p65 translocation to the nucleus in a PPARγ-dependent manner. In Chagas disease, both the sustained increment in pro-inflammatory mediators and microvascular abnormalities are crucial aspects for the generation of cardiac damage. Elucidating the mechanism of action of new PPARγ ligands is highly attractive, given the fact that it can be used as an adjuvant therapy, particularly in the case of Chagas disease in which inflammation and tissue remodeling play an important role in the pathophysiology of this disease.

## Introduction

*Trypanosoma cruzi (T. cruzi)* is a protozoan parasite that causes Chagas disease, the main cause of infectious dilated cardiomyopathy all over the world (1). This vector-borne disease affects millions of people in South America and, in recent years, it has been regarded as a risk factor for transfusion and vertical transmission in countries without vector-borne disease transmission control. During the acute stage, inflammation is involved in protection but parasite persistence leads to chronic inflammation. This impairs adequate repair leading to cumulative damage that ultimately may cause death due to cardiac insufficiency (2). Likely, chronic Chagas cardiomyopathy (CCC) is the most important clinical manifestation of Chagas disease. Clinical manifestations are characterized by conduction system disturbances, atrial and ventricular arrhythmias, congestive heart failure, systemic and pulmonary thromboembolism, and microvascular dysfunction (3). Although the mechanisms underlying progression to CCC have not been fully understood, it is generally accepted that inflammation persistence plays a predominant role (4).

Depending on their levels, oxidative species (mainly H_2_O_2_) can promote cell redox signaling or cytotoxicity (5). *T. cruzi* infection, together with pro-inflammatory cytokines, H_2_O_2_ and NO production by cardiac, endothelial, and immune cells, leads to an increase in nitroxidative stress that may account for host cell and tissue damage (6–8).

In response to the infection, monocytes can differentiate into macrophages and are one of the main infiltrating leukocytes to reach the myocardium earlier (9). These cells play important roles in the infection outcome and are essential for the orchestration of immunity and cardiac homeostasis. Due to their functional and phenotypic versatility, manipulating specific macrophage subsets can be crucial in collaborating with vital cardiovascular functions, such as tissue repair and defense against the infection (10). PPARγ are key nuclear receptors and therapeutic targets for the treatment of metabolic diseases through the regulation of insulin resistance, diabetes, and dyslipidemia (11). Moreover, in recent decades, it has been shown that PPARγ and its ligands can repress inflammatory genes in activated macrophages (12–14) and *T. cruzi*-infected cardiomyocytes (15, 16), including the inducible NO synthase (iNOS), cyclooxygenase 2 (COX2), IL-6, and TNF-α, via the nuclear factor kB (NF-κB) pathway. Although a few drugs that target PPARγ, such as troglitazone, rosiglitazone, and pioglitazone, have been approved for different pathologies, severe adverse effects have led to discover diverse and novel compounds that target PPARγ (17). In this regard, Brun et al. synthesized a new PPARγ agonist with potent anti-inflammatory properties and without cytotoxic activity, tested in macrophages stimulated with LPS, and in a murine model of dextran-induced colitis (18). This compound is a 2,4-substituted 3-hydroxy-4-pyridinecarboxylic acid derivative (HP_24_), an aza-analog of salicylic acid and structurally close to other potent anti-inflammatory pyridine compounds, such as aminopyridinylmethanols and aminopyridinamines. In a recent work, we demonstrated that HP_24_ interacts with PPARγ in *T. cruzi*-infected macrophages, using a molecular docking approach. Also, we showed that treatment with a HP_24_ increased the expression of pro-angiogenic molecules, like endothelial NO synthase (eNOS) and VEGF-A, inhibited pro-inflammatory mediators, and reduced fibrosis in the heart of *T. cruzi*-infected mice (19). Furthermore, it has been reported that PPARγ agonists improve the vascular function, which is partially dependent on eNOS activation through the AKT pathway (20–22). In the context of *T. cruzi* infection, PI3Kγ signaling in myeloid cells restricts heart parasitism and avoids heart damage and death of mice (23). Moreover, along the infection, PI3K/AKT signaling activation is able to prevent infected cells from dying by repressing the apoptotic machinery (24). The aim of this work is to deepen into the mechanisms involved in the pro-angiogenic and anti-inflammatory effects of HP_24_ in *T. cruzi*-infected macrophages, as they have not yet been elucidated. Our results reveal that HP_24_ increases pro-angiogenic mediators (eNOS and VEGF-A) through PI3K/AKT/mTOR and PPARγ signaling. Lastly, we provide, for the first time, evidence that HP_24_ inhibits reactive nitrogen and oxygen species production and the NF-κB pathway by PPARγ-dependent mechanisms in peritoneal macrophages from *T. cruzi*-infected mice.

## Materials and Methods

### Ethics Statement

To carry out this work, BALB/c mouse were bred and maintained in the animal facility at the Department of Microbiology, Parasitology and Immunology, School of Medicine, University of Buenos Aires. All the procedures were approved by the Institutional Committee for the Care and Use of Laboratory Animals (CICUAL, School of Medicine, University of Buenos Aires, CD N° 2271/2014), in line with guidelines provided by the Argentinean National Administration of Drugs, Food and Medical Devices (ANMAT), the Argentinean National Service of Sanity and Agrifoods Quality (SENASA), and also based on the US NIH Guide for the Care and Use of Laboratory Animals.

### Mice and Infection

All mice were provided with a 12-h day/night cycle and water and fed *ad libitum* with a standard diet. Seven male mice *per* group were infected intraperitoneally with 1 × 10^5^ bloodstream trypomastigotes of a lethal RA (pantropic/reticulotropic) subpopulation of *T. cruzi* (25). Euthanasia was carried out by CO_2_ inhalation at 9 days post-infection (dpi). Each experiment was performed at least three times.

### Synthesis of 1-Methyl-3-Hydroxy-4-Pyridinecarboxylic Acid Derivative 24 (HP_24_)

1-Methyl-3-hydroxy-4-pyridinecarboxylic acid derivative was resynthesized following the pathway reported previously by Brun et al. (18), with some modifications in the reaction conditions for the final steps of the synthesis and the purification step that led to the desired compound HP_24_ in the zwitterions form instead of the chloride compound described above. 3-Hydroxy-isonicotinic acid (1 g, 7.18 mmol) was suspended in 5 ml of DMF in a 25-ml round-bottomed flask. The resulting suspension was stirred at room temperature, and 10% NaOH (7.5 ml) was added dropwise until complete dissolution of the solid (pH 9–10). Methyl iodide (2.06 g, 14.46 mmol, *d* = 2.28 g/ml, 0.9 ml) was added under stirring and the solution was then refluxed, monitoring the reaction progress by thin-layer chromatography (*n*-butanol:H_2_O:AcOH, 1:1:1). Once the starting material disappeared, the solvent was removed under reduced pressure, obtaining a deep orange colored solid, which was dissolved in boiling water (50 ml). The solution was acidified with 37% HCl (3.5 ml), and 10% H_2_O_2_ (1 ml) was added. Then, the iodine was exhaustively extracted with CHCl_3_ (5 × 15 ml) in a separating funnel. The organic phase was concentrated under pressure to dryness, obtaining an orange crude raw powdery solid (1.662 g), which was purified by reversed-phase chromatography in a Biotage Isolera Spektra Flash Chromatography apparatus equipped with prepacked C18 cartridges. The fractions containing the product were pooled and concentrated to dryness by means of a rotary evaporator, yielding a white powdery product (0.956 g, 6.21 mmol).

### 3-Hydroxy-1-Methylpyridin-1-Ium-4-Carboxylate (HP_24_)

Yield: 86.4%; mp: 236°C (decomposition); R_f_: 0.13 (*n*-butanol:H_2_O:AcOH, 1:1:1); IR (KBr): ν (cm^−1^) = 3.432 (OH), 3.079 (= C–H), 2.850 (CH_3_), 1.654 (COO–), 1.480 (C = C), 1.381 (C = N), 1.300 (C–N) cm^−1^; ^1^H NMR (300 MHz, [D6] DMSO) δ = 8.43 (s, 1H, H-2), 8.01 (d, *J* = 6.00 Hz, 1H, H-6), 7.98 (d, *J* = 6.03 Hz, 1H, H-5), 4.19 ppm (s,3H, N-CH_3_); ^13^CNMR (75 MHz, [D6] DMSO) δ = 47.98 (N-CH3), 126.85 (C-5), 129.73 (C-4), 130.72 (C-6), 137.54 (C-2), 164.99 (C-3), 166.95 ppm (COO–); HRMS (ESI-MS, 140 eV): *m*/*z* [M^+^H^+^] calculated for C_7_H_7_NO_3_+, 154.0504; found, 154.0545; RT-HPLC, C18: *t*_R_ = 5.40 min, 97.61 A%; elemental analyses: calculated for C_7_H_7_NO_3_, C 54.9%, H 4.61%, N 9.15%; found: C 54.47%, H 4.39%, N 8.98%.

### Isolation of Peritoneal Macrophages

Macrophages were obtained by washing the peritoneal cavity of uninfected or *T. cruzi*-infected mice (9 dpi) with 8 ml of RPMI-1640 culture medium (Invitrogen Life Technologies, Grand Island, NY, USA), supplemented with 10% of heat-inactivated fetal bovine serum (FBS) (Internegocios S.A., Argentina) and antibiotics (50 μg/ml of PenStrep^®^). Cells were allowed to adhere to the plastic surface of 6-well culture plates (Greiner Bio One International AG) for 3 h at 37°C in a 5% CO_2_ atmosphere (26). For PPARγ silencing experiments using PPARγ siRNA and Oligofectamine^®^, cells were cultured up to 30% confluence with RPMI-1640 medium, without FBS and PenStrep^®^.

### *In vitro* Treatment With HP_24_ and LY294002

Peritoneal macrophages were isolated from uninfected or *T. cruzi*-infected mice (9 dpi) as indicated above. Cells were treated *in vitro* with HP_24_ (100 μM in PBS) or the specific PI3K inhibitor LY294002 (30 μM in DMSO) (Sigma-Aldrich Co., St. Louis, USA). For *in vitro* experiments, treatments were performed 30 min prior to infection. After the different treatments, cell viability was examined by a Trypan blue dye exclusion test.

### PPARγ Knock-Down With Small-Interfering RNA (siRNA)

Macrophages were cultured up to 30–50% of confluence in RPMI-1640 medium without FBS and antibiotics for 24 h. Thereafter, cells were transfected with 20 μM of either of two different Stealth siRNA (7863 or 7864, Invitrogen Life Technologies, Grand Island, NY, USA) that target PPARγ mRNA, following the manufacturer's instructions. Transfections were performed with Oligofectamine, as specified by the manufacturer. Assays for gene activity were performed at 72 h post-transfection. The impact of PPARγ-siRNA interference on PPARγ mRNA was evaluated by RT-qPCR. PPARγ Stealth siRNA sequence: PPARGMSS-7863 forward: 5′-CCAGGAGAUCUACAAGGACUUGUAU-3′, reverse: 5′-AUACAAGUCCUUGUAGAUCUCCUGG-3′; PARGMSS-7864 forward: 5′-UCAAGGGUGCCAGUUUCGAUCCGUA-3′, reverse: 5′-UACGGAUCGAAACUGGCACCCUUGA-3′.

### RNA Purification

Total RNA was obtained from macrophages using Quickzol reagent (Kalium Technologies, Argentina), treated with RQ1 RNase-Free DNase (Promega Co., USA). Total RNA was reverse-transcribed using M-MLV Reverse Transcriptase (Promega Co., USA), according to the manufacturer's instructions.

### Quantitative Reverse Transcription Polymerase Chain Reaction (RT-qPCR)

mRNA expression was determined using 5 × HOT FIREPOL EVAGREEN qPCR (Solis BioDyne, Estonia) in a StepOnePlus Real-Time PCR System. Parameters were as follows: 52°C for 2 min, 95°C for 15 min, and 40 cycles at 95°C for 15 s, 54°C (for PPARγ) or 60°C (for 18S) for 30 s and 72°C for 1 min. Normalization was carried out using 18S rRNA. Quantification was performed using the comparative threshold cycle (Ct) method, as all the primer pairs (target gene/reference gene) were amplified using comparable efficiencies (relative quantity, 2^−ΔΔCt^) (27, 28). Primer sequences: 18S forward: 5′-AACACGGGAAACCTCACCC-3′, reverse: 5′-CCACCAACTAAGAACGGCCA-3′; PPARγ forward: 5′-ATCTACACGATGCTGGC-3′, reverse: 5′-GGATGTCCTCGATGGG-3′.

### Protein Extraction and Western Blot Analysis

Total and cytosolic protein extracts were prepared as described previously by our group (19, 29). Protein concentration was determined by the Bradford method using a commercial protein assay (Bio-Rad, USA) and bovine serum albumin (BSA, Sigma-Aldrich Co, USA) as a standard (30). Fifty micrograms of protein extracts separated in 8–12% SDS-PAGE gels was blotted onto a Hybond-P membrane (GE Health-care, Spain) and incubated with the following specific antibodies: anti-PPARγ (Santa Cruz Biotechnology, CA, USA; Cat#sc-7273), anti-phospho-AKT (Ser 473) (Biolegend Inc., USA; Cat#649001), anti-AKT (Biolegend Inc., USA; Cat#680302), anti-phospho-p70S6K (Ser 411) (Santa Cruz Biotechnology, CA, USA; Cat#sc-8416), anti-p70S6K (Santa Cruz Biotechnology, CA, USA; Cat#sc-8418), anti-VEGF-A (Santa Cruz Biotechnology, CA, USA; Cat#sc-1836), anti-eNOS (Santa Cruz Biotechnology, CA, USA; Cat#sc-654), anti-iNOS (Santa Cruz Biotechnology, CA, USA; Cat#sc-650), anti-phospho-IKK (Ser 180) (Santa Cruz Biotechnology, CA, USA; Cat#sc-23470), anti-IKK (Santa Cruz Biotechnology, CA, USA; Cat#sc-7606), anti-IκB-α (Santa Cruz Biotechnology, CA, USA; Cat#sc-371), and anti-α-actin (Santa Cruz Biotechnology, CA, USA; Cat#sc-1615). All specific antibodies were diluted 1:500 in PBS. Blots were revealed by enhanced chemoluminescence in a BioSpectrum^®^ Imaging System (UVP, Analytik Jena Company, USA). Band intensity was analyzed using the NIH Image J software (ImageJ).

### Immunocytochemistry and Confocal Laser Microscopy Imaging

Macrophages were grown on round glass coverslips and fixed with methanol and blocked with 3% BSA in PBS. The expression of iNOS was determined by immunofluorescence, as described previously (31). The expression of p65 was determined by confocal microscopy. For these purposes, rabbit polyclonal IgG anti-iNOS (Santa Cruz Biotechnology, CA, USA; Cat#sc-650), rabbit polyclonal IgG anti-p65 (Santa Cruz Biotechnology, CA, USA; Cat#sc-109), and a rabbit polyclonal IgG directed to *T. cruzi* developed in our laboratory were used as primary antibodies at a 1:50 dilution, and goat anti-rabbit IgG Alexa Fluor 488 nm (for iNOS) (Jackson ImmunoResearch Labs; Cat# 111-545-003) or goat anti-rabbit IgG Alexa Fluor 647 nm (for p65 and *T. cruzi*) (Jackson ImmunoResearch Labs; Cat# 111-605-003) was used at a 1:500 dilution as secondary antibodies. The coverslips mounted with DAPI-Fluoromount-G (SouthernBiotech) were examined under a confocal microscope (ZEISS LSM800) using a Plan Apochromat 63× 1.42 numerical aperture (NA) oil immersion objective or under an Eclipse Ti-S fluorescence microscope (Nikon) using a Plan Apochromat 100 × 1.42 NA oil immersion objective. To quantify parasitism, the percentage of infected cells and the number of parasites *per cell* were determined by analyzing the presence of intracellular amastigotes and trypomastigotes in at least 30 random microscopic fields. Mean fluorescence intensity (MFI) were quantified using the Fiji version of the open source Image J software (NIH, USA) (32).

### NO Measurement

To determine the amount of NO released into the culture medium, nitrate was reduced to nitrite and measured spectrophotometrically using the Griess reaction (33, 34). The amount of NO in culture supernatants was calculated by interpolation of the samples absorbance at 540 nm using a standard curve of NaNO_2_.

### Detection of H_2_O_2_ Generation

Macrophages were plated onto 96-well polystyrene plates, up to 30% confluence, and silencing of PPARγ was performed. After 72 h, macrophages were pre-treated with HP_24_ and infected with *T. cruzi*. Cellular H_2_O_2_ production was measured using a DCFDA assay kit (Abcam) according to the manufacturer's protocol. DCFDA (2′,7′-dichlorofluorescein diacetate), a cell-permeable fluorogenic dye, is deacetylated by cellular esterases to a non-fluorescent compound, and later oxidized by ROS to highly fluorescent 2′,7′-dichlorofluorescein (DCF), which measures peroxyl, hydroxyl, and other ROS activities within the cell. DCF can be detected by fluorescence spectroscopy with maximum excitation and emission spectra of 495 and 529 nm, respectively. After 24 h post-infection, cells were washed twice with PBS, incubated with 25 μM DCFDA in RPMI medium at 37°C for 45 min, and evaluated by flow cytometry using a FACSCanto (BD Biosciences). The percentage of positive cells and MFI were analyzed with FlowJo X software (TreeStar).

### Statistical Analysis

Data are expressed as the mean of three independent experiments ± SEM (*n* = 3) (six mice/group) for each experimental group. One-way ANOVA was used to analyze the statistical significance of the differences observed between infected, treated, and untreated groups. The Tukey *post-hoc* test was performed to compare every mean with every other mean. Differences were considered statistically significant when *P* < 0.05. All analyses were performed using the Prism 7.0 Software (GraphPad Prism).

## Results

### Neither HP_24_ nor PPARγ Modifies Parasitism in *T. cruzi*-Infected Macrophages

**Figure 1:**
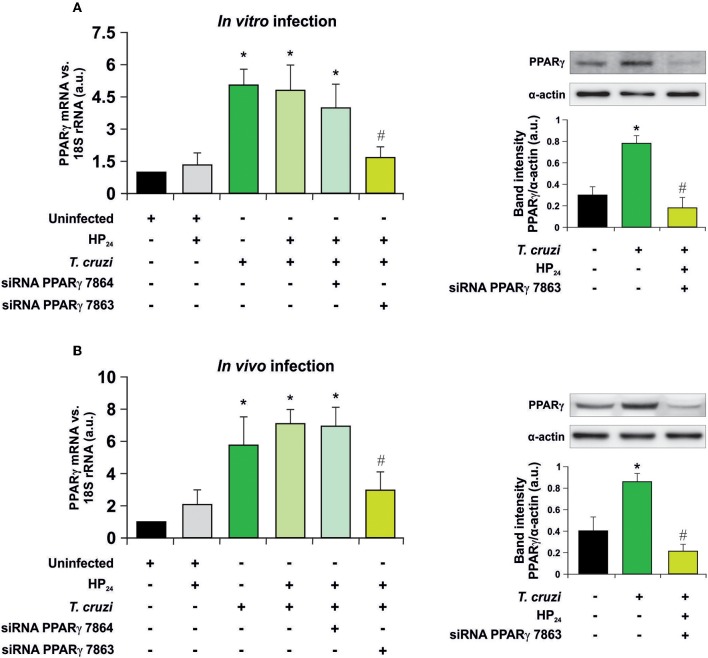
PPARγ silencing. **(A)** Uninfected peritoneal macrophages were obtained and, alternatively, transfected with two different PPARγ-siRNA during 72 h. Transfected or non-transfected cells were treated with HP_24_ (100 μM) since 30 min before infection. These cells were infected for 48 h with *T. cruzi* (parasite:cell ratio 5:1). **(B)** Peritoneal macrophages from *T. cruzi*-infected mice (9 dpi) were obtained. These cells were alternatively transfected with two different PPARγ-siRNA for 72 h. Transfected or non-transfected cells were treated with HP_24_ (100 μM) for 48 h. PPARγ expression was analyzed by RT-qPCR (six mice/group). Expression of PPARγ was determined by Western blot, and protein levels were normalized against α-actin. For both models, RNA and proteins were isolated 48 h after HP_24_ treatment. Results represent the mean ± SEM of three independent experiments ^*^P < 0.05 vs. uninfected cells, ^#^*P* < 0.05 vs. *T. cruzi*-infected cells.

Recently, we have shown that treatment with HP_24_ affects neither parasitemia nor the number of amastigote nests in the heart of infected mice (19). To test whether signaling of HP_24_ through PPARγ correlates with changes in cell parasitism, we performed silencing assays to knock down PPARγ. First, the silencing effectiveness of PPARγ siRNA 7863 and 7864 was verified in peritoneal macrophages infected *in vitro* with *T. cruzi* (parasite:cell ratio 5:1) (Figure 1A), as well as in peritoneal macrophages from *T. cruzi-*infected mice (9 dpi) (Figure 1B). RT-qPCR assays revealed that, in both models, the maximum level of silencing was obtained with siRNA 7863 (70–60% of silencing). To confirm these results, we performed Western blot assays and determined that PPARγ siRNA 7863 was effective in silencing the expression of this receptor, and thus this was the siRNA used for further studies (Figures 1A,B).

**Figure 2:**
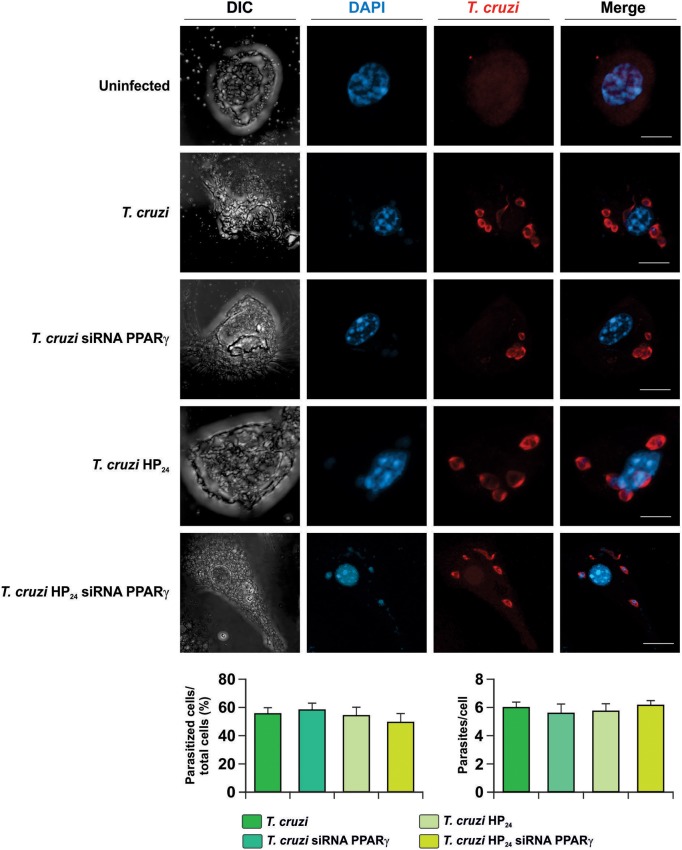
Neither HP_*24*_ nor PPARγ modifies parasitism in *T. cruzi*-infected macrophages. Peritoneal macrophages were obtained and transfected with PPARγ-siRNA during 72 h. Transfected or non-transfected cells were treated with HP_24_ (100 μM). Then, these cells were infected with *T. cruzi* for 48 h (parasite:cell ratio 5:1). *T. cruzi* was detected by confocal microscopy with a rabbit polyclonal anti-*T. cruzi* antibody and a secondary goat anti-rabbit Alexa 647-labeled antibody. Cell nuclei were stained with DAPI. Representative microphotographs are shown. The percentage of infected cells and the number of parasites *per cell* were determined by analyzing the presence of intracellular amastigotes and trypomastigotes in at least 30 random microscopic fields. Scale bar: 5 μm. Results represent the mean ± SEM of three independent experiments.

Next, the involvement of HP_24_ and PPARγ in the possible changes in parasite load was evaluated. To this aim, isolated macrophages were infected *in vitro* with *T. cruzi* and the percentage of infected cells and the number of parasites per cell were evaluated by confocal microscopy. We observed that neither HP_24_ nor PPARγ silencing affects the parasitism of macrophages (Figure 2).

### HP_24_ Regulates VEGF-A and eNOS Expression by PI3K/AKT/mTOR and PPARγ Signaling in *T. cruzi*-Infected Macrophages

**Figure 3:**
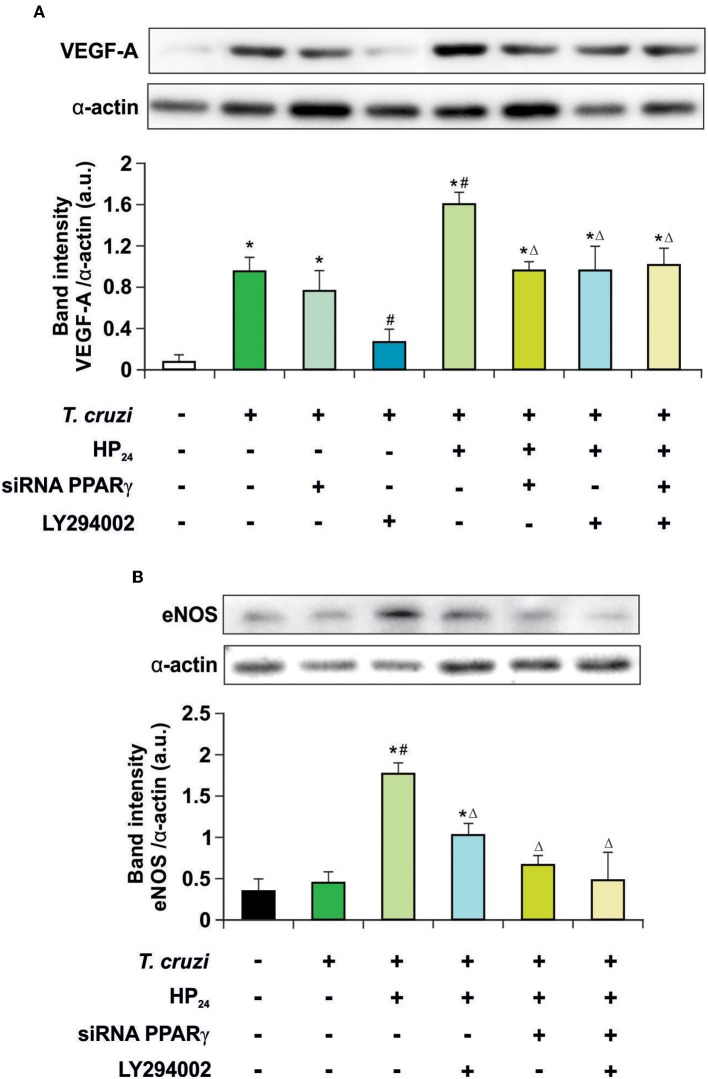
HP_24_ regulates eNOS and VEGF-A expression by PPARγ and AKT signaling pathway. Peritoneal macrophages from *T. cruzi*-infected mice (9 dpi) were obtained. Cells were transfected with PPARγ-siRNA during 72 h. Transfected or non-transfected cells were treated with HP_24_ (100 μM) or HP_24_ (100 μM) plus LY294002 (30 μM) for 48 h. **(A,B)** Expression of eNOS and VEGF-A was determined by Western blot, and protein levels were normalized against α-actin. Results represent the mean ± SEM of three independent experiments (six mice/group). ^*^P < 0.05 vs. uninfected cells, ^#^*P* < 0.05 vs. *T. cruzi*-infected cells. Δ*P* < 0.05 vs. *T. cruzi*-infected HP_24_-treated cells.

Recently, we described that a new PPARγ ligand, HP_24_, increases the expression of VEGF-A and eNOS in *T. cruzi*-infected macrophages. These effects were prevented in the presence of T0070907, a specific PPARγ antagonist (19), suggesting ligand-dependent activity. It has been widely reported that the PI3K/AKT/mTOR pathway plays a prominent role in regulating angiogenesis (35). Therefore, the involvement of the PI3K/AKT/mTOR and PPARγ pathways in the regulation of VEGF-A and eNOS expression by HP_24_ was analyzed in *T. cruzi*-infected macrophages. Cells were infected with *T. cruzi* and either treated with LY294002 (PI3K inhibitor) or transfected with PPARγ-siRNA, or both. Infection of macrophages augmented expression of VEGF-A but not eNOS (Figures 3A,B). To test whether the PPARγ and PI3K/AKT/mTOR pathways are involved in the increased expression of VEGF-A in infected cells, PPARγ was silenced or treated with LY294002. In the absence of the receptor, no changes in the expression of VEGF-A were observed. Moreover, inhibition of the PI3K signaling impeded the increase of VEGF-A. This suggests that the PI3K/AKT/mTOR pathway is associated to the elevation of VEGF-A induced by *T. cruzi* infection. Then, the effect of HP_24_ on VEGF-A and eNOS expression was evaluated. The PPARγ ligand significantly increased the expression of both pro-angiogenic mediators in macrophages. The participation of PPARγ was demonstrated, since HP_24_ could not increase pro-angiogenic mediators in infected and transfected cells. Moreover, upon PI3K inhibition with LY294002 of *T. cruzi-*infected cells, HP_24_ could not increase both pro-angiogenic mediators. Lastly, when PPARγ-siRNA transfected cells were pre-incubated with LY294002 and treated with HP_24_, similar results were observed for eNOS and VEGF-A. These results suggest independent, though not necessarily additive, pro-angiogenic effects of HP_24_ involving the PI3K/AKT/mTOR and PPARγ pathways (Figure 3).

**Figure 4:**
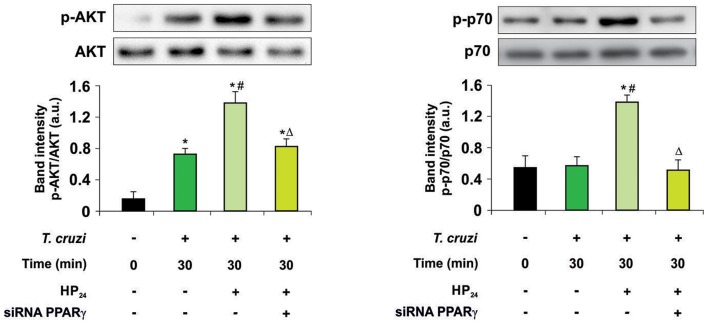
PPARγ is required for the HP_24_ effects on PI3K/AKT/mTOR signaling pathway. Peritoneal macrophages were obtained and transfected with PPARγ-siRNA during 72 h. Transfected or non-transfected cells were treated with HP_24_ (100 μM) since 30 min before infection. Then, these cells were infected with *T. cruzi* for 30 min (parasite:cell ratio 5:1). Western blot analyses were carried out in cytosolic extracts, and p-AKT (Ser 473)/total AKT and p-p70 (Ser 411)/total p70 expression was analyzed. Results represent the mean ± SEM of three independent experiments. ^*^P < 0.05 vs. uninfected cells, ^#^*P* < 0.05 vs. *T. cruzi*-infected cells. Δ*P* < 0.05 vs. *T. cruzi*-infected and HP_24_-treated cells.

Then, we kept delving into the participation of PPARγ in the effect of HP_24_ on the PI3K/AKT/mTOR pathway. For this purpose, we analyzed AKT and the ribosomal protein S6 kinase (p70S6K) phosphorylation, since they are considered to be the hallmark of the PI3K/AKT/mTOR pathway activation. Macrophages were treated with HP_24_ (100 μM) for 45 min before *T. cruzi* infection (parasite:cell ratio 5:1). Increased phosphorylation of AKT but not p70S6K was observed in macrophages after 30 min of infection (Figure 4). When infected macrophages were treated with HP_24_, AKT phosphorylation increased further and p70S6K phosphorylation showed significant phosphorylation in comparison with infected untreated cells (Figure 4). PPARγ silencing prevented the effect of HP_24_ on the phosphorylation of AKT and p70S6K. As expected, phosphorylation of AKT and p70S6K was inhibited in cells treated with LY294002 (data not shown). These results indicate that PPARγ is required for the effects of HP_24_ to take place on the PI3K/AKT/mTOR pathway (Figure 4).

### HP_24_ Inhibits Reactive Nitrogen and Oxygen Species in *T. cruzi*-Infected Macrophages Through PPARγ

**Figure 5:**
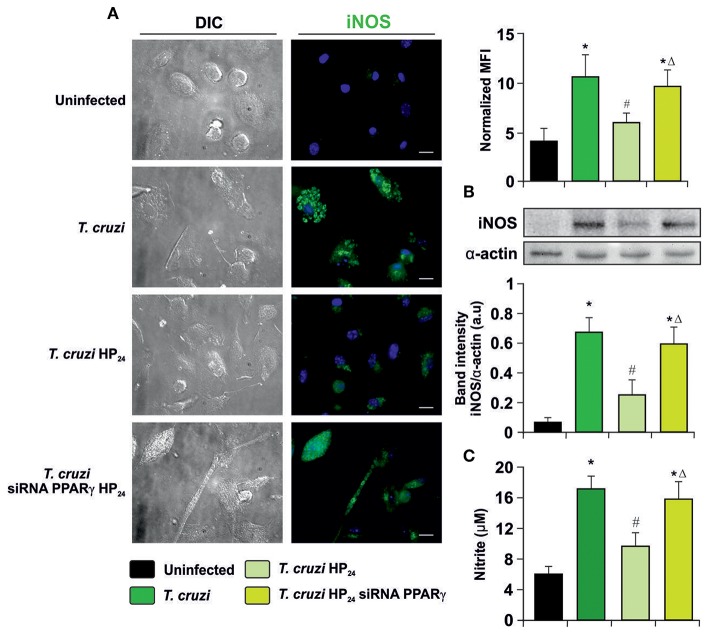
Participation of PPARγ in iNOS inhibition by HP_24_. **(A)** Peritoneal macrophages from *T. cruzi*-infected mice (9 dpi) were obtained. Cells were transfected with PPARγ-siRNA during 72 h. Transfected or non-transfected cells were treated with HP_24_ (100 μM) for 48 h. iNOS expression was detected by immunofluorescence with a rabbit polyclonal anti-iNOS antibody and with a secondary goat anti-rabbit Alexa 488-labeled antibody. Cell nuclei were stained with DAPI. Mean fluorescence intensity (MFI) represents iNOS expression. Representative microphotographs are shown. Scale bar: 10 μm. **(B)** Western blot analysis was carried out and iNOS expression was determined. Protein levels were normalized against α-actin. **(C)** NO release to culture supernatants was analyzed by the Griess method. Results are expressed as the mean of three independent experiments (six mice/group). ^*^P < 0.05 vs. uninfected cells, ^#^*P* < 0.05 vs. *T. cruzi*-infected cells. Δ*P* < 0.05 vs. *T. cruzi*-infected HP_24_-treated cells.

To determine whether HP_24_ modulates iNOS expression and activity through PPARγ, immunocytochemistry (ICQ) analysis was performed as well as Western blot assays and detection of NO release by the Griess reaction on *T. cruzi*-infected macrophages. iNOS expression was significantly increased in infected macrophages in comparison with uninfected ones. Moreover, HP_24_ treatment inhibited iNOS expression, while it was restored in PPARγ siRNA-transfected cells (Figure 5A). Western blot analysis confirmed these findings, since iNOS expression increased upon infection, decreased after treatment with PPARγ agonist and this decline was reversed in silenced cells (Figure 5B). Lastly, the activity of iNOS, as evidenced by the release of NO, paralleled the expression of iNOS observed by ICQ and Western blot (Figure 5C).

**Figure 6:**
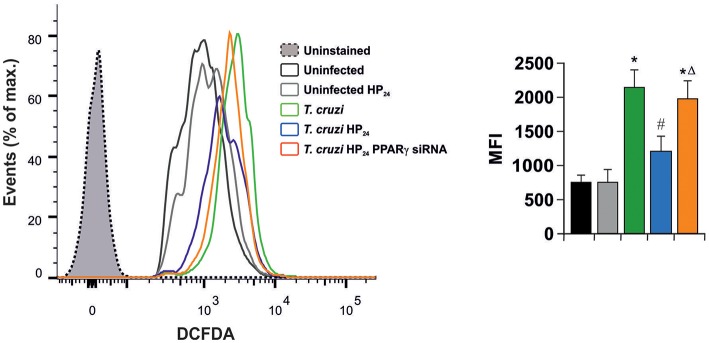
HP_24_ inhibits H_2_O_2_ production through PPARγ in *T. cruzi*-infected macrophages. Peritoneal macrophages were obtained and transfected with PPARγ-siRNA for 72 h. Transfected or non-transfected cells were treated with HP_24_ (100 μM) since 30 min before infection. Then, these cells were infected with *T. cruzi* for 24 h (parasite:cell ratio 5:1). Sample histogram shows H_2_O_2_ generation measured by flow cytometry using 2′,7′dichlorofluorescein-diacetate (DCFDA). Ten thousand events were acquired for each group. Data are presented as the mean MFI ± SEM of three independent experiments. ^*^P < 0.05 vs. uninfected cells, ^#^*P* < 0.05 vs. *T. cruzi*-infected cells. Δ*P* < 0.05 vs. *T. cruzi*-infected HP_24_-treated cells.

In light of these results, we aimed to determine whether HP_24_ affects H_2_O_2_ production (reactive oxygen species). Macrophages were infected *in vitro* with *T. cruzi* for 24 h. A high content of H_2_O_2_ was observed, as measured by flow cytometry, using the redox-sensitive fluorescent probe, 2′,7′-dichlorofluorescein-diacetate (DCFDA). HP_24_ treatment significantly inhibited H_2_O_2_ production. Moreover, when PPARγ was silenced, HP_24_ could not exert this effect in *T. cruzi*-infected cells. These results suggest that HP_24_ modulates H_2_O_2_ production through PPARγ (Figure 6).

### HP_24_ Inhibits the NF-κB Pathway by PPARγ-Dependent Mechanisms

**Figure 7:**
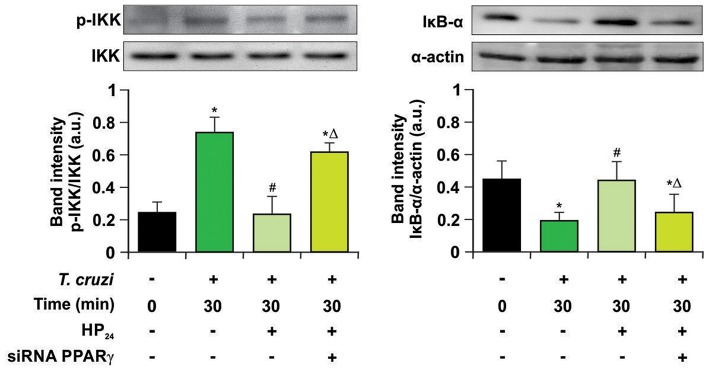
HP_24_ inhibits IKK and IκB-α phosphorylation by PPARγ-dependent mechanisms. Peritoneal macrophages were obtained and transfected with PPARγ-siRNA for 72 h. Transfected or non-transfected cells were treated with HP_24_ (100 μM) since 30 min before infection. Then, these cells were infected for 30 min with *T. cruzi* (parasite:cell ratio 5:1). Cytosolic expression of p-IKK (Ser 180) and IκB-α was determined by Western blot, and protein levels were normalized against IKK total and α-actin. Results are expressed as mean of three independent experiments ^*^P < 0.05 vs. uninfected cells, ^#^*P* < 0.05 vs. *T. cruzi*-infected cells. Δ*P* < 0.05 vs. *T. cruzi*-infected HP_24_-treated cells.

Previously, we demonstrated that 15dPGJ2, a natural ligand of PPARγ, is a potent modulator of the inflammatory process through PPARγ-dependent and -independent pathways in *T. cruzi*-infected cardiac cells (15, 16). In this work, we analyzed whether the new PPARγ ligand HP_24_ exerts its anti-inflammatory effects through the NF-κB pathway in a PPARγ-dependent manner. For that aim, two different approaches were designed: Firstly, we evaluated the effects of HP_24_ on IKK and IκB-α, two cytosolic components of NF-κB pathway, by Western blot. HP_24_ treatment inhibits IKK phosphorylation and IκB-α degradation in *in vitro* infected *T. cruzi* macrophages. Moreover, in PPARγ-silenced cells, HP_24_ was unable to exert its inhibitory effect on both components (Figure 7).

**Figure 8:**
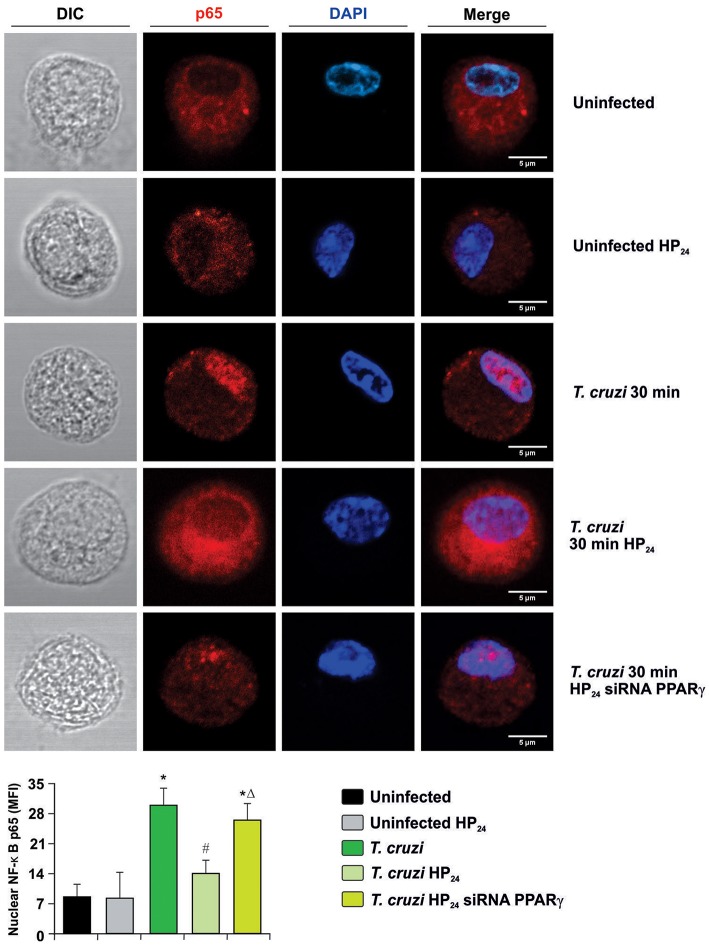
HP_24_ inhibits the NF-κB pathway by PPARγ-dependent mechanisms. Peritoneal macrophages were obtained and transfected with PPARγ-siRNA for 72 h. Transfected or non-transfected cells were treated with HP_24_ (100 μM) since 30 min before infection. Then, these cells were infected for 30 min with *T. cruzi* (parasite:cell ratio 5:1). p65 expression was detected by confocal microscopy with a rabbit polyclonal anti-p65 antibody and a secondary goat anti-rabbit Alexa 647-labeled antibody. Cell nuclei were stained with DAPI. Representative microphotographs are shown. MFI represents nuclear p65. Scale bar: 5 μm. Results are expressed as the mean MFI of three independent experiments. ^*^P < 0.05 vs. uninfected cells, ^#^*P* < 0.05 vs. *T. cruzi*-infected cells. Δ*P* < 0.05 vs. *T. cruzi*-infected HP_24_-treated cells.

Secondly, we measured the translocation of p65 subunit of NF-κB to the nucleus by confocal microscopy in PPARγ-silenced cells. Figure 8 shows that p65 translocates to the nucleus at 30 min after infection, and HP_24_ treatment inhibits this translocation. However, under silencing conditions, HP_24_ was unable to inhibit NF-κB activation, confirming that HP_24_ regulates NF-κB pathway in a PPARγ-dependent manner (Figure 8).

## Discussion

This work describes for the first time the signaling pathways of HP_24_, a new synthetic PPARγ ligand, in *T. cruzi*-infected macrophages. This compound is derived from 3-hydroxy-4-pyridinecarboxylic acid (18). Although we recently published that HP_24_ has important pro-angiogenic and anti-inflammatory properties (19), the signaling cascades involved in these effects have not been studied yet.

In this work, we demonstrate that treatment with HP_24_ does not modify parasite load, in comparison with infected untreated cells. This is in agreement with previous results from our group using HP_24_ using an *in vivo* model of acute infection (19). Also, we showed similar results using a PPARα ligand, in an *in vivo* model of chronic infection (29). Besides, Rodrigues et al. show reduction of skeletal muscle parasitism (36).

Macrophages infected with *T. cruzi* and treated with HP_24_ display activation of the PI3K/AKT/mTOR and PPARγ signaling pathways, leading to induction of VEGF-A and eNOS expression. Consistent with this, it has been described that pioglitazone treatment up-regulates VEGF-A and eNOS expression, significantly ameliorates endothelial dysfunction, and enhances blood flow recovery after tissue ischemia in diabetic mice, via AKT phosphorylation (37). Moreover, it has been shown that rosiglitazone restores endothelial dysfunction in a rat model of metabolic syndrome, through PPARγ- and PPARδ-dependent phosphorylation of eNOS and AKT (38). In this study, we show that HP_24_ can regulate the PI3K/AKT/mTOR signaling pathway at 30 min in a PPARγ-dependent manner in *T. cruzi*-infected macrophages. Moreover, our results show that in PPARγ-siRNA transfected cells, HP_24_ is unable to increase phosphorylation levels of AKT and p70S6K, strongly suggesting a link between PI3K/AKT/mTOR signaling and the role of PPARγ as a transcription regulator.

Recognition of the pathogens by resident and recruited macrophages activates several signaling pathways, including PI3K signaling. This pathway is involved in different cellular processes, such as cytoskeletal rearrangement, membrane trafficking, and endosome fusion through the phosphorylation of lipids and proteins (39, 40). Regarding *T. cruzi*, several authors have demonstrated that infection activates PI3K signaling in human and mouse macrophages leading to increased infection and anti-apoptotic pathways that allow for intracellular parasite multiplication and survival (24, 41, 42). Recently, it has been demonstrated that canonical PI3Kγ signaling in myeloid cells is essential to restrict *T. cruzi* heart parasitism and ultimately to avoid myocarditis, heart damage, and death in mice (23). Furthermore, in a human *in vitro* model of osteoarthritis characterized by increased advanced glycation end (AGE) products accumulation and reduced autophagy, it was demonstrated that pioglitazone, a PPARγ ligand, increased AKT/mTOR phosphorylation in a dose-dependent manner, leading to better cell viability and inducing increased chondrocyte autophagy (43). However, several works on cancer and neurodegenerative diseases have shown that PPARγ activation inhibits the PI3K/AKT/mTOR signaling pathway (44–48). Likewise, it has been described that the PI3K/AKT/mTOR-p70S6K activation pathway is critical in restricting pro-inflammatory and promoting anti-inflammatory responses in TLR-stimulated macrophages (49).

We have previously shown that different PPAR ligands are potent inhibitors of inflammatory mediators like NO, IL-1β, IL-6, and TNFα, through PPAR-dependent and -independent pathways in *T. cruzi*-infected macrophages and cardiomyocytes (15, 16, 26, 29). In the present study, we determined the anti-inflammatory efficacy of HP_24_. We observed that this new compound inhibits the production of H_2_O_2_ and the release of NO by reducing iNOS expression in a PPARγ-dependent manner in *T. cruzi*-infected macrophages. It is well-documented that the activation of the respiratory burst of macrophages in response to infection with *T. cruzi* inflicts oxidative damage to host tissues. ROS and nitric oxide (NO) combine to form peroxynitrite, participating in the destruction of parasites phagocytosed by activated macrophages (50). Nevertheless, if the parasites are not completely eliminated, this phagocytic cell response may persist in the chronic stage of the infection and contribute to oxidative damage that impairs heart function (51). It has been reported that PPARγ ligands induce anti-inflammatory effects, mainly mediated by antioxidant properties. PPARγ activation by β3-adrenergic receptor inhibits the activation of the NADPH oxidase and leads to the expression of catalase in macrophages and myometrial cells in an *in vitro* model of preterm labor (52). Furthermore, the role of PPARγ in the protection of muscle fibers against oxidative stress caused by excessive acute exercise in Sprague–Dawley rats has been demonstrated (53). Moreover, Liu Z. and coworkers established that PPARγ alleviates oxidative stress and adipose inflammation by binding to Mark4 promoter region in Kunming male mice (54). Over the past decade, several authors have demonstrated that during the early acute phase, ROS production could favor *T. cruzi* infection in macrophages (8, 55, 56). Based on these results, it could be suggested that HP_24_ affects the course of infection, since it inhibits the production of H_2_O_2_ and NO. However, in this work, we demonstrate that HP_24_ treatment does not affect the parasite load. These results are in agreement with a recent work where we showed that HP_24_ treatment affects neither parasitemia nor the number of amastigote nests in the heart of acutely infected mice (19).

It has been widely demonstrated that NF-κB is involved in the pro-inflammatory response in different models of *T. cruzi* infection (15, 29, 31, 57, 58). This transcriptional factor regulates the expression of several pro-inflammatory cytokines and chemokines such as TNFα, IL-1β, IL-6, and pro-inflammatory gene expression such as iNOS, COX2, and metalloproteases, among others (59). Several authors have shown that different PPARγ ligands exert anti-inflammatory effects through the inhibition of NF-κB-dependent inflammatory genes (60–63). In the same line of evidence, we demonstrated the anti-inflammatory properties of several PPAR ligands in different models of *T. cruzi* infection (15, 16, 29). Herein, we evidenced that HP_24_ inhibits NF-κB activation in *T. cruzi*-infected macrophages. Moreover, we showed that knockdown of PPARγ by specific siRNA impedes the HP_24_-mediated inhibition of NF-κB in macrophages infected with *T. cruzi*. These findings indicate the specificity of the HP_24_ effects and the inhibition of NF-κB in a PPARγ-dependent manner.

In Chagas disease, both the sustained increase in pro-inflammatory mediators as a result of parasite persistence and microvascular abnormalities are crucial aspects in the generation of cardiac damage. In this context, elucidating the mechanism of action of new PPARγ ligands as an alternative to thiazolidinediones is highly attractive since they may serve as possible adjuvants in anti-inflammatory therapy in combination with anti-parasitic treatments used currently, to avoid or delay irreversible tissue damage in the host.

**Figure 9:**
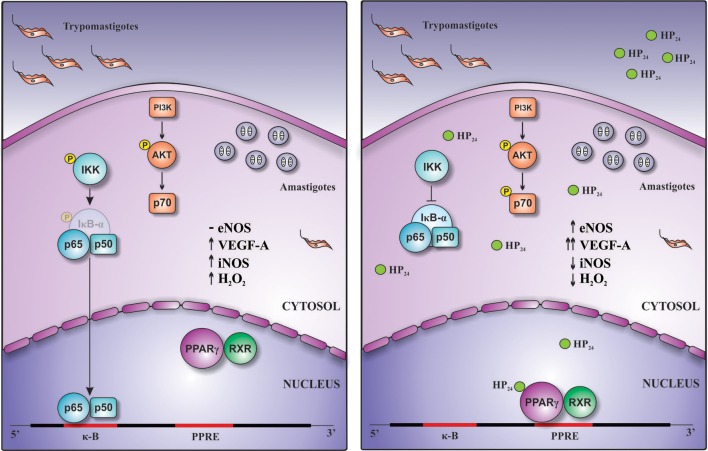
Schematic representation of the pro-angiogenic and anti-inflammatory actions of HP_24_ in *T. cruzi*-infected macrophages. Treatment of *T. cruzi*-infected macrophages with HP_24_ exerts pro-angiogenic effects, increasing the expression of eNOS and of VEGF-A. This effect depends on both PPARγ and PI3K/AKT/mTOR pathways. Besides, HP_24_ avoids activation of NF-κB, thereby preventing iNOS expression, NO release, and H_2_O_2_ production. This anti-inflammatory effect occurs in a PPARγ-dependent manner.

Overall, this work demonstrates for the first time that this new PPARγ ligand, HP_24_, exerts anti-inflammatory and pro-angiogenic effects through PI3K/AKT/mTOR and PPARγ signaling (Figure 9).

## Data Availability Statement

All datasets generated for this study are included in the article.

## Ethics Statement

The animal study was reviewed and approved by the Institutional Committee for the Care and Use of Laboratory Animals (CICUAL, School of Medicine, University of Buenos Aires, CD N° 2271/2014).

## Author Contributions

NG and FP designed the experiment. FP, NG, ÁC, and GM contributed to the writing of the manuscript. FP, ÁC, MR, and AP did experiments. FP, NG, and GM analyzed the data. MF and DC provided the 3-hydroxy-4-pyridinecarboxylic acid derivative (HP_24_). NG, MS, GM, and FP contributed to the final approval of the version to be published.

### Conflict of Interest

The authors declare that the research was conducted in the absence of any commercial or financial relationships that could be construed as a potential conflict of interest.
